# Study on preventing adverse events in neonates (SEPREVEN): A stepped-wedge randomised controlled trial to reduce adverse event rates in the NICU

**DOI:** 10.1097/MD.0000000000020912

**Published:** 2020-07-31

**Authors:** Laurence Caeymaex, Cecile Lebeaux, Jean Christophe Roze, Claude Danan, Audrey Reynaud, Camille Jung, Etienne Audureau

**Affiliations:** a Faculty of Health and CEDITEC, University Paris East Creteil; b Neonatal Intensive Care Unit, Centre Hospitalier Intercommunal de Creteil; c Clinical Research Center (CRC), Centre Hospitalier Intercommunal de Creteil, Créteil; d Pediatric Intensive Care Unit Nantes, University Hospital Centre Nantes, Pays de la Loire; e Association SOS Prema, Boulogne Billancourt; f IMRB INSERM U 955 Team CEpiA (Clinical Epidemiology and Ageing Unit), Creteil, Val de Marne; g Assistance Publique Hôpitaux de Paris (APHP), Hôpital Henri-Mondor, Clinical Research Unit (URC), Public Health Department, Créteil, France.

**Keywords:** adverse events, communication, medical errors, neonatal intensive care units, neonates, safety

## Abstract

**Introduction:**

Adverse events (AE) in care are recognized as a leading cause of mortality and injury in patients. Improving patients’ safety is difficult to achieve. Therefore, innovative research strategies are needed to identify errors in subgroups of patients and related severity of outcomes as well as reliably measured efficiency of reproducible strategies to improve safety. This trial aims to evaluate the impact of a combined multiprofessional education program on the rate of AE in neonatal intensive care units (NICUs).

**Methods and analysis:**

This is a stepped-wedge cluster randomised controlled trial with 3 clusters each containing 4 units. The study time period will be 20 months. The education program will be implemented within each cluster following a random sequence with a control period, a 4-month transition period and a post-educational intervention period. Eligibility criteria: for clusters: 6 NICUs from Ile-de-France and 6 NICUs from different regions in France; for patients: in-hospital during the study period (November 23, 2015 and November 2, 2017 [inclusion start dates varying by unit]) in one of the 12 NICUs; corrected gestational age ≤42 weeks upon admission; hospitalization period >2 days; and parents informed and not opposed to the use of their newborn's data. A routine occurrence reporting of medical errors and their consequence will take place during the entire study period. The intervention will combine an education to implement a standardized root cause analysis method, creation of bundles (insertion, daily goals, maintenance bundles) to prevent catheter-associated blood-stream infection and a poster to prevent extravasation injuries. Outcome: We hypothesize a reduction from 60 (control) to 50 (intervention) AE/1000 patient-days. The primary outcome will be the rate of AE/1000 patient-days in the NICU.

**Trial registration number:**

NCT 02598609, trial registered November 6, 2015. https://clinicaltrials.gov/ct2/show/NCT02598609.

**Ethics and dissemination:**

Study approved by the regional ethic committee CPP Ile-de-France III (no 2014-A01751-46). The results will be published in peer-reviewed journals.

## Abstract 2

Supplemental Digital Content is available in the text

## 1 Introduction

Since 1999, medical errors (MEs) have been recognized as a leading source of mortality in patients.^[1]^ Recent reports suggest that as many as 250,000 to 400,000 patients die each year in the United States as a result of MEs and that millions of preventable injuries occur per year due to MEs.^[2]^ MEs have been defined as unintended acts in the process of care, either omission or commission, or acts that do not achieve their intended outcome.^[3,4]^ Most MEs do not result in harm to the patient, but a proportion of them causes harm^[5,6]^; these MEs are called preventable adverse events (AEs) – injuries or damages resulting from a medical intervention or omission of an intervention. Consequences of AEs vary in severity, from treatment modifications to contributing to death.^[2,5,7]^

Common risk factors for AEs are invasive procedures, long hospital stays^[8–11]^ and organizational factors such as professionals’ turnover, night/day shifts, and complex transmissions.^[12,13]^

Neonates hospitalized in neonatal intensive care units (NICUs) are at high risk of ME-related damage. In this population, rates of AEs vary by degree of prematurity: the risk of AEs is 57% for extremely low-gestational-age (GA) neonates born before 28 weeks’ GA (ELGANs) as compared with 3% for term newborns.^[9]^ Supplementary risk factors in the ELGAN subgroup are clinical fragility due to low weight, overall immaturity and inability to compensate for clinical alterations as well as complex care (catheters, tubes, artificial ventilation) and types of medications needed.^[9,14–21]^ In addition, neonatal-specific AEs described include identification errors among multiple-birth infants,^[22]^ breast milk errors,^[21]^ skin lesions due to immaturity^[9,15]^ and nasal injuries from non-invasive ventilation.^[23]^ Finally, hospital-acquired infections (HAIs), especially central-line-associated bloodstream infections (CLABSIs) are a leading cause of mortality and neurological morbidity in premature neonates^[23–25]^ which can be reduced by adherence to appropriate infection control measures.^[26,27]^

Various strategies exist to measure patient safety in clinical settings. To measure MEs and AEs rates over time, trigger-based retrospective chart reviews (RCRs) are the method of choice. These systematic medical-record surveillance methods are sensitive and reliable, detecting AEs at 10-fold higher rates than administrative screening tools and hospital reporting systems.^[28,29]^ Voluntary and anonymous occurrence reports by healthcare professionals (HCPs; nurse, resident or physician) may help identify MEs and near misses but are unreliable to measure the evolution of ME and AE rates over time because they depend on HCPs’ perseverance and regularity to collect them exhaustively, and HCPs may miss some MEs and AEs such as HAIs.^[28–30]^

In the last decades, patient safety efforts have focused on harm rather than error.^[31]^ However, the results of studies are disappointing, because reducing AE rates seems difficult to achieve.^[5,32]^ Besides checklists^[33]^ and bundles^[34]^ to prevent some types of HAI, a common approach to prevent the recurrence of AEs is to proceed to a review of care, such as root cause analysis (RCA), an analysis of triggers by a sentinel event that resulted in damage (physical or psychological injury) or death. RCA aims at identifying the cause(s) that underlie variations in performance and thus improving the systems and processes to decrease the odds of an event.^[35]^ Since the 1990s, various RCA approaches have been used, and the lack of standardization of these methods has resulted in a variation in the quality and comprehensiveness of the outcomes. In the last years, some problems have been described with RCA,^[36]^ as well as a lack of strong evidence to support its effectiveness in improving safety in medical settings.^[37–40]^

This study aims to evaluate the impact of combined multi-professional education on the rate of AEs in NICUs. This program combines education in a standardized RCA-like method^[41]^ and the creation of bundles (insertion bundle, daily goals bundle, maintenance bundle) to prevent late-onset neonatal central-line-associated bloodstream infection. The outcome is measured by RCR based on a preexisting specific NICU trigger tool method.^[42]^

This is a stepped-wedge cluster randomised trial (SW-CRT) with trigger-based RCR measurement of AE rates in neonates hospitalized in NICUs. The stepped-wedge design relies on the sequential implementation of the experimental intervention within all participating units during subsequent periods and in a randomized order.^[43,44]^ In contrast to more conventional parallel-group cluster randomized trials, all clusters will benefit at some point from the intervention of interest, thus accumulating information both during a pre- and post-interventional period (each cluster being its own control). Two main situations typically justify the use of a stepped-wedge design: firstly, cases in which an intervention is deemed globally beneficial with limited drawbacks, ethically justifying its wide implementation in all participating clusters and/or secondly, cases in which organizational, logistical or financial reasons preclude immediate and simultaneous implementation in all units randomized to the experimental group. For the present trial, the following elements supported the decision to use a stepped-wedge design: interest of the intervention potentially beneficial for all centres; practical interest of the progressive implementation of the intervention; statistical interest related to a potentially more effective control of intra-cluster correlation allowing, under certain conditions, a statistical power greater than that of conventional cluster trials at equivalent numbers of people^[45]^; interest in monitoring over time the persistence of the expected benefit and to detect possible underlying “natural” temporal trends; and interest in obtaining balance in cluster characteristics after randomization, in particular when the number of clusters is low.

## 2 Methods and analysis

### 2.1 Study design

**Figure 1:**
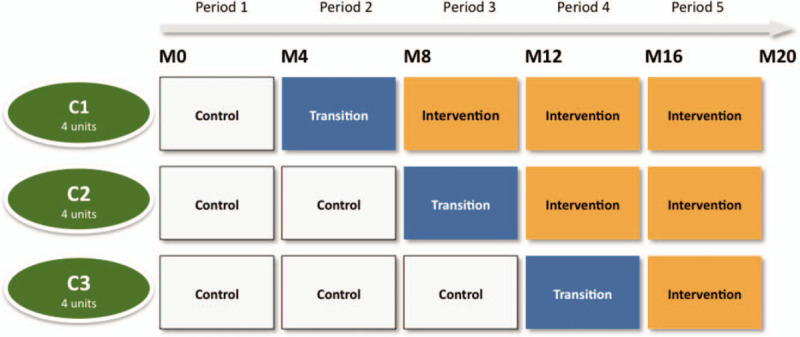
Diagram of the timing of the intervention in the SEPREVEN stepped-wedge cluster randomised controlled trial. The trial lasts 20 months (M) per participating unit. Each randomization cluster (C) C1, C2 and C3 comprises 4 units. Intervention is implemented within each randomization cluster following a random sequence. The gray cells represent the (pre-educational) *control* periods, blue cells a 4-months *transition* phase and orange cells the (post-educational) *intervention* periods. The transition phase allows for the time to integrate the program into the units. During this *transition* period the unit is not in a *control* or *intervention* phase and does not contribute to primary endpoint analysis.

This is an SW-CRT. The study diagram (Fig. 1) presents the timing of the interventions and the steps per cluster.

Sample selection: Study population and setting

Patient eligibility criteria

Inclusion criteria: All patients will be included as follows:

- In hospital during the 20-month study period between November 23, 2015 and November 2, 2017 (inclusion start dates varying by unit) in one of the 12 participating units,
- Corrected GA ≤ 42 weeks (≤42 weeks + 6 days) at the time of admission,
- Hospitalization period is longer than 2 calendar days,
- Parents have been informed and will not oppose the use of their newborn's data.

### 2.2 Participating units’ eligibility criteria

The following criteria were considered to include hospitals in this study: NICUs should exclusively be dedicated to newborn care (no pediatric ICU); should not have planned to implement a new ordering program; and for feasibility reasons and external validity, 6 hospitals should be located in Ile de France (Paris area) and 6 in distinct regions in France.

### 2.3 Recruitment of patients

The investigators at each site will be responsible for including all the patients who meet the inclusion criteria. The study will be proposed to parents, and the information note signed by the investigating doctor will be delivered to them; another copy will be kept by the investigator for 15 years. To optimize recruitment in this routine care study, the information letter will be delivered to parents by the HCP usually in charge of patient care. In the absence of parental opposition, the infant will be included in the study.

### 2.4 Randomization and allocation concealment

Randomization will be performed at the unit-level (no randomization oat the patient level). Before the trial starts, the units will be randomized to one of the 3 sequences (4 units/cluster, see Fig. 1) by use of a computer-generated list, without pre-specified characteristics. Each cluster will receive the intervention at different times: therefore, the total duration of the 3 periods will not be the same for the 12 units. Time blocks of 4 months will be considered, including the individual control and intervention periods as well as the transient phase.

### 2.5 Intervention

The program itself consists of 3 educational actions to be implemented during the 4-month “transition” period.

At the local unit-based level, 3 actions will take place:

First, it will consist of training in the ORION standardized RCA method, derived from aeronautics and adapted for several years to care.^[41]^ This method allows local HCPs to perform an RCA in a simple way in their NICU setting. At the beginning of the intervention period, local HCPs (NICU senior physicians and nurses, optionally a risk manager, maximum 13 persons) will be trained in situ for 1 day to conduct a feedback meeting and perform a systemic analysis of risky events. Following this training, the RCA will be implemented from the transient phase on; each of the RCAs will consist of a chronological reconstruction of the facts, leading to the collective identification of one or more barrier actions to be implemented and a verification of the effective implementation of the actions decided in the preceding RCA. This training will be delivered once; a second 4-hour training will be implemented if asked by the unit. The training is meant to be used during the months following the transient phase.

Second, the intervention will include training for each unit in preventing central-line-associated bacteraemia with the participation of a local senior NICU physician and nurses involved in patient care: each unit will create bundles (insertion bundle, daily goals bundle, maintenance bundle) according to the rules of good practice for central venous catheters promoted internationally,^[26]^ with a possible adaptation to specific local practices. These bundles are meant to be used continuously during the intervention period.

Third, a poster created for the study will present the drugs and solutions at risk of necrosis in the event of extravasation through the peripheral venous route. This poster is meant to be used continuously during the intervention period.

The anticipated effect of this multifaceted intervention is a mixture of immediate and delayed effects: for the RCA education, a delayed but sustained effect regarding various AEs, and for bundles and the extravasation poster, an immediate and sustained effect.

At the cluster level, 2 telephone meetings will be held by the investigator (LC) during the transition phase, as well as guidance and follow-up of the creation of bundles.

### 2.6 Outcomes

#### 2.6.1 Primary endpoint and primary outcome measure

The primary objective of the study is the effectiveness of a multiprofessional training program for reducing the rate of AEs measured by RCR with a NICU trigger tool. The corresponding primary endpoint will be the rate of AEs/1000 patient-days in the NICU, compared between control and intervention (post-educational) periods with the SW-CRT design.

#### 2.6.2 Secondary endpoints and secondary outcome measures

1. Rates of preventable AEs/1000 patient-days. Assessment of the effectiveness of the intervention in reducing the rates of preventable AEs (number of AEs/1000 patient-days) measured by RCR.
2. Rates of severe and non-severe AEs/1000 patient-days.^[46]^ Assessment of the effectiveness of the intervention in reducing the rates of severe and non-severe AEs (number of severe and non-severe AEs/1000 patient-days) measured by RCR.
3. Rates of CLABSIs/1000 patient-days and /1000 catheter-days. Assessment of the effectiveness of the intervention in reducing the rates of CLABSIs measured by RCR.
4. Rates of HAIs/1000 patient-days. Assessment of the effectiveness of the intervention in reducing the rates of HAIs measured by RCR.
5. Rates of unprogrammed extubations requiring reintubation/1000 patients-days and /100 ventilator days and /100 hours. Assessment of the effectiveness of the intervention in reducing the rates of these unplanned extubations measured by RCR.
6. Rates of medication errors/1000 patient-days. Assessment of the effectiveness of the intervention in reducing the rates of medication errors measured by RCR.
7. Description of types and severity of MEs and AEs occurring in NICU patients with a prospective rating of severity of outcome after the AE occurred by using the National Coordinating Council for Medication Error Reporting and Prevention (NCCMERP) classification index. Description will be based on routine care occurrence reports by HCPs for the entire study population.
8. Economic impact measured by using the entire study population's clinical data and outcome criteria and impact of the intervention measured by using the RCR population's clinical data and outcome criteria.
9. Health-related outcomes impact of the study (measured by using the entire study population’ outcome criteria) and impact of the intervention (measured by using the RCR population's outcome criteria).
10. Description of severe extravasation injuries and evolution (context and rate per patients).
11. Description of bacterial species collected from blood culture specimens: genotype, phenotype and resistance to antimicrobial and antiseptic agents

For units participating in these secondary objectives:

11. Prospective dermatitis score before and after the use of antiseptic for central line insertion in premature neonates born before 32 weeks’ GA and <15 days old.
12. Description of nasal scores during the use of non-invasive nasal ventilation in premature neonates born before 32 weeks’ GA.
13. HCPs’ attitude regarding AE disclosures to parents. Description of characteristics of disclosed and undisclosed AEs, HCPs’ motivations, and perceived parental reactions. Description will be based on a prospective self-administered questionnaire for HCPs for each routine care occurrence report.
14. Impact of the number of nurses and patients present in the NICU on rates of AEs. Prospective collection of the number of nurses and number of patients according to their level of care.
15. Impact of each unit's preexisting safety culture on the impact of the intervention.

### 2.7 Data collection

#### 2.7.1 Record-review process for the primary endpoint

This part of the data collection will start during 2018, after the end of the patients’ inclusion period, and will imply to involve external reviewers, not present in the prior steps of the trial.

Record reviews (RCRs) will be conducted by using the NICU-specific trigger tool.^[42]^ It will be applied according to the procedure described in the NICU Trigger Toolkit^[47]^ translated into French by our research team for the purpose of this study. This NICU trigger tool consists of 14 triggers or clues that indicate the possibility of AE-induced harm. According to this method, when the examiner finds a trigger, she/he examines the record further to determine whether the injury apparently resulted from an AE. As mentioned in the NICU Trigger Toolkit, AEs identified via sources other than the trigger are also considered. To capture only AEs related to the participating NICU, AEs already present on admission and infections occurring before day 2 after admission to the unit will not be considered AEs. For each record, the reviewers will complete a table corresponding to the triggers found and the potential AEs. With an additional document, they will record on an AE collection form the standardized information for each identified potential AE (name, preventability, NCCMERP-graded severity^[46]^ occurrence date, comment, associated triggers). All potential AEs and associated characteristics will be confirmed or not by a physician reviewer.

#### 2.7.2 Routine care occurrence reports over the 20-month period

This part of the data collection will take place prospectively during the 20-month inclusion period. A routine voluntary multiprofessional reporting of MEs and consequence will take place to identify MEs and AEs. Routine nasal injury scores and skin scores will also be completed during this entire period. HAIs will be recorded by a NICU physician on dedicated forms. The disclosure procedures for communicating the MEs and AEs to parents will be detailed in an additional form in the units participating in this study component.

#### 2.7.3 AE characterization: severity and preventability

The NCCMERP classification index will be used to rate the severity of the MEs and AEs.^[46]^ This index has the advantage of standardizing the damage severity classification, thus allowing to compare data from studies. According to this classification, lower-severity harms are defined as category E and higher-severity harms as categories F, G, H or I. This index will be used for the routine care occurrence reports and for the severity grading of RCR AEs.

Preventable AEs will be defined as any event that could have been avoided by an appropriate error management strategy.^[48]^ According to these proposals, HAIs, most MEs and unplanned extubations would be considered preventable. Other AEs such as skin lesions or nasal injuries would need an individual assessment to determine preventability. In case of uncertainty regarding an AE's severity and/or preventability, an expert neonatologist committee, blinded to the AE's occurrence period, will be asked to conclude.

#### 2.7.4 Other characteristics

Demographic data (birth date, sex, multiple gestation, etc.), medical data (gestational age at birth, birth weight, ventilation, central lines, etc.), and outcomes (mortality and specific outcomes) as well as ME and AE data (nature, severity of consequences, communication) and HAI data (dates, antibacterial treatment duration, data on blood cultures and local bacterial specimens) will be collected for all patients.

### 2.8 Statistical analysis plan

#### 2.8.1 Sample size and power calculation

The primary endpoint is the rate of AEs/1000 patient-days (hospitalization days). Analysis of available data from the literature as well as our own unpublished routine data allowed for anticipating an average basal value of 60 AEs/1000 patient-days in the absence of any intervention. The calculation of the required sample size was based on the Poisson distribution to compare control and intervention AE rates. To do so, calculations were performed according to Hemming and Girling,^[49]^ derived from the initial approach by Hussey and Hughes,^[50]^ to take into account the effect of the stepped wedge (or design effect) study specification on the sample size calculation.

Considering a total number of 12 units divided into 3 randomization clusters over 5 study periods, a 5% two-tailed alpha risk and an intraclass correlation coefficient ranging from 0.01 to 0.1, we will need to include 15 patients per unit per month (i.e., 15 × 4 = 60 patients per cluster per 4-month evaluation period), for a total of 15 × 20 × 12 = 3600 patients over the study period, a cumulative 61,200 to 72,000 patient-days corresponding to an expected mean hospitalization length from 17 to 20 days) to show, with a power of 80%, a minimum relative reduction of 17% in rates of AEs (i.e., a decrease from 60 [control] to 50 [intervention] AEs/1000 patient-days).

#### 2.8.2 Statistical analysis

Stepped-wedge cluster trials present a number of challenges related to intra-cluster correlation, the correlation between data repeated over time, and the need to monitor underlying temporal trends.^[47]^ Accordingly, the analysis of the primary endpoint (AEs/1000 patient-days) and other event rate secondary endpoints (e.g., number of AEs/100 admissions, preventable AEs/1000 hospital days etc.) will be based on mixed-effects Poisson regression models, entering as fixed effects the control period and a time term to account for a potential underlying trend in AE rates over the study period, and a random effect accounting for the unit level. A potential interaction term between time and intervention will be tested to identify a possible learning curve.^[5]^ Pre-specified supportive analyses will be performed to take into account possible changes in specific confounding factors over time by further adjusting for the demographic and clinical characteristics of children, including a lower term – GA at birth <28 weeks; birth weight <1000 g; length of hospital stay.^[9,19,25]^ Treatment-effect heterogeneity across clusters and varying secular trends across clusters will also be investigated by introducing the intervention and time terms as random slopes, respectively.^[51]^

The analysis of binary secondary endpoints will be based on mixed-effects logistic regression models, following similar modelling principles such as those previously described for the primary endpoint.

The descriptive analysis results will be presented as number (%) for categorical data or mean (standard deviation) or median (interquartile range) for continuous variables, depending on the characteristics of the observed distributions. Univariate comparisons between periods will rely on Student's *t* test for paired series or Wilcoxon signed ranks for quantitative variables, depending on the application conditions.

All missing or invalid data will be systematically checked and searched for in patients’ medical records. For multivariate analysis of the primary endpoint adjusting for potentially missing confounders, in addition to complete case analysis, a sensitivity analysis will be led using multiple imputation by chained equations to check the robustness of the findings

All analyses will be performed with Stata v16.0 (StataCorp, College Station, TX, USA).

#### 2.8.3 Outcomes measured

The effectiveness of the intervention will be measured by RCR of files selected by drawing lots. As previously mentioned, this analysis will involve using the NICU trigger tool developed and described by Paul Sharek.^[42,47]^ Fifteen files/month/unit will be drawn at random from the pool of all patient files included in the study, for each of the 20 months of participation. Only the part of the file corresponding to the stay in the unit participating in SEPREVEN will be considered. AEs will be assigned to the control or intervention period according to their date of occurrence.

The records will be analyzed by reviewers external to the unit and to the promotor, educated in the RCR for the study purpose. The reviewers will be unaware of the study intervention and clusters. The analysis will be made progressively in alphabetical order of identification code, thus mixing files from the different periods. To limit internal incoherence in analysis, the same reviewer will analyze all charts from a same unit. Each suspected AE will have to be confirmed by a physician educated in the RCR and unaware of the patients’ dates of stay, in agreement with the external reviewer. In the event of disagreement between the reviewer and the physician about the presence, name, severity or preventability of the AE, the case will be discussed with a third physician and consensus will be sought.

Once validated, the trigger-tool data and AE data will be entered in the e-CRF.

### 2.9 Ethics, trial management and dissemination

Trial registration: NCT, registration number NCT 02598609, registered November 6, 2015.

https://clinicaltrials.gov/ct2/show/NCT02598609

The SEPREVEN study was approved by the National Data Protection Authority (CNIL no 915263) and the ethics committees (Consultative Committee on the Treatment of Data on Personal Health for research Purposes, France CCTIRS no 15327) and Committee for the Protection of People Participating in Biomedical Research CPP Ile-de-France III, France (no ID RCB: 2014-A01751-46).

A four-letter number will be automatically generated when a new patient is entered on the e-CRF. A correspondence sheet containing this number and the patient's identity (surname, first name, date of birth) will be completed by the research assistant of each unit. At the e-CRF level, all data will be anonymized. The data recorded during this research will be processed electronically in accordance with Act No. 78-17 of 6 January 1978 on data processing, files and liberties, as amended.

Finally, as part of the study, each unit's team will sign a commitment to respect a specific Caregiver Protection Charter, which implies the requirement of non-punitiveness of HCPs associated with error reporting in the study.

Protocol contributors: LC conceptualised the study, EA conceptualised the design and analysis. CJ is responsible for the project administration.

The results will be published in peer-reviewed journals and disseminated through presentation at scientific conferences. Authors will be professionals participating in the research.

### 2.10 Funding

This work was supported by a grant from the French Solidarity and Health Ministry, Programme de Recherche sur la Performance du Système des soins (PREPS) Grant no. 13-0401. The funder had no role in the design and conduct of the study, the collection, analysis or interpretation of data or writing of the manuscript.

### 2.11 Competing Interests

The authors LC, JCR, CJ; CD, EA, AR and CL have declared that no financial competing interests exist. The AFM42 Company that provided the RCR-like educational program (ORION method) as part of the intervention, had no role in study design, data collection and analysis, decision to publish, or preparation of the manuscript.

### 2.12 Protocol amendments

The research will be conducted in accordance with this protocol. Protocol amendments with substantial changes in the protocol have been tracked and dated. Up to January 2020, 4 amendments have been submitted and have been accepted by the Committee for the Protection of People Participating in Biomedical Research CPP Ile-de-France III - no ID RCB: 2014-A01751-46). Content of substantial changes and regulatory authorities agreement are available as a supplementary file.

### 2.13 Patient and public involvement statement

Patient representatives (Association SOS Prema, France) were not involved in the protocol design, but will be partners of the research in the reporting of the results and outcomes and in the dissemination plans. They will also be involved in the study on communication of errors with parents.

### 2.14 Monitoring

In the first months of the study, a monitoring will cover at least 10 files per unit and will concern all data to be entered for these files. Additional monitoring will be carried out on the units for which anomalies have been identified as well as for all the patients who died. Another specific monitoring will concern AE collection (date, type, severity).

A global monitoring will be generated over time automatically from the eCRF-level to complete or correct missing or irrelevant data and sent to the units until correction is made. LOS data will be monitored by comparing the data of each unit with those of its annual institutional report of bacteriaemia. Regarding implementation of the intervention, there will be no direct audit procedure, but an independent company is responsible for the RCA-like educational program in the units. For the second part of the intervention (bundles) the principal investigator (LC) and the local investigator will work together in order to create, print and implement the bundles.

Regarding the data from the trigger tool RCR, all the charts will be analyzed by professionals from another independent company and checked by a clinician.

## 3 Discussion

Patient safety is a key issue that concerns everyone using the healthcare system but also carers, policy makers, health economists, and the whole society. Despite this, at this stage, the rate of AEs does not seem to be controllable, and studies measuring the efficiency of standardized prevention programs are lacking. In comparison with studies of other health-related causes of death, studies improving knowledge in this field are rare. As commented by Peerally et al, RCA presents a number of flaws – like susceptibility to political hijack, tendency to produce poor risk controls, poorly functioning feedback loops, failure to aggregate learning across incidents and confusion about blame and responsibility.^[36,37,40]^ Innovative context-related research strategies are needed to identify preventable AEs, related severity of outcomes as well as a reliable measure of the efficiency of strategies to improve safety.^[2]^

Typically, AEs are types of injuries that most frequently are due to an error in treatment rather than the underlying condition of the patient. However, in clinical practice, preventability definition is not consensual because of a degree of subjectivity and context-dependent causalities.^[48,52]^ A systematic review in 2012 concluded no available definition of preventability; the most common definition used in studies was the presence of an identifiable and modifiable cause of harm, completed with the concept of a failure to act according to “available knowledge” or to follow “accepted practices” on a system or individual basis.^[48]^ Some authors have proposed to create an internationally accepted definition of preventable AEs to increase the reproducibility of RCRs and the identification of ME-related injuries.^[53]^

Our study will generate knowledge in the field of patient security by targeting 2 dimensions: first, it will assess the efficiency of an educational program combining prevention of nosocomial infections and a type of RCA targeting also other types of AE, in a NICU micro-environment. Second, it will offer a prospective aggregate description of MEs’ characteristics in NICU patients as well as the damage they caused at the multi-institutional level. The grading of severity will help identify the AEs most involved in patients’ altered security. Hence, our project combines actions related to patient safety in its various components. Finally, our data will raise some original ethical issues around the disclosure of MEs to families and on the proxy's role as a co-actor in patient safety.

## Author contributions

**Conceptualization:** Laurence Caeymaex, Jean-Christophe Rozé, Claude Danan, Camille Jung, Etienne Audureau.

**Data curation:** Laurence Caeymaex, Camille Jung.

**Formal analysis:** Laurence Caeymaex, Etienne Audureau.

**Funding acquisition:** Laurence Caeymaex.

**Methodology:** Laurence Caeymaex, Jean-Christophe Rozé, Claude Danan, Camille Jung, Etienne Audureau.

**Project administration:** Camille Jung.

**Software:** Laurence Caeymaex, Camille Jung.

**Supervision:** Laurence Caeymaex, Jean-Christophe Rozé.

**Validation:** Jean-Christophe Rozé, Audrey Reynaud.

**Writing – original draft:** Laurence Caeymaex, Cecile Lebeaux, Etienne Audureau.

**Writing – review & editing:** Laurence Caeymaex, Cecile Lebeaux, Jean-Christophe Rozé, Claude Danan, Audrey Reynaud, Camille Jung.

## Supplementary Material

Supplemental Digital Content
