# Supplementary material for: Study on preventing adverse events in neonates (SEPREVEN): A stepped-wedge randomised controlled trial to reduce adverse event rates in the NICU
Source: Medicine (Baltimore). 2020 Jul 31;99(31):e20912. doi: 10.1097/MD.0000000000020912 (PMC7402760; doi:10.1097/MD.0000000000020912)
Supplement: Supplemental Digital Content [file medi-99-e20912-s001.pdf]

COMITE DE PROTECTION DES PERSONNES
ILE DE FRANCE III
Hôpital Tarnier-Cochin, 89 rue d'Assas 75006 Paris - Tel : 01.46.33.68.67 - Fax : 01.46.33.70.46
Email : cpp.iledefrance3@orange.fr

Composition du bureau **: Président : David SIMHON, Vice-Président : Denis BERNARD, Secrétaire : Nadine LABBE, Trésorier : Paulette MORIN.**

A Paris, le 27 mars 2015

DS/LG/2015-052

**REF** : A rappeler dans toute correspondance<br>Dossier n°: 2014-A01751-46<br>Réf. CPP : S.C. 3229

Le Comité a été saisi le 08 décembre 2014 d'une demande d'avis pour un projet de recherche de soin courant intitulé : **SEPREVEN** : ***«Etude sur la prévention et l'épidémiologie des évènements indésirables en réanimation - soins intensifs néonatals » - «Study on Prevention and Epidemiology of adverse events in neonates » ;***

dont le Gestionnaire est : Centre hospitalier intercommunal de Créteil ;

et le responsable est : Laurence CAEYMAEX .

La recherche aura lieu au sein des centres investigateurs suivants : CHI Créteil, Hospices Civils de Lyon, CHU Hautepierre (Strasbourg), CHU Bicêtre (Le Kremlin-Bicêtre), CHU Caen, CHI Poissy, CHU Robert Debré (Paris), CH Saint-Denis, CHU Jeanne de Flandres (Lille), CHU Angers, CHU Grenoble.

Le Comité a notamment examiné le protocole de la recherche –version 1.0 du 21/11/2014 -, le formulaire d'information et de non opposition – version 1.0 du 21/11/2014 -, ainsi que tous les autres documents communiqués par le promoteur et a tenu compte de la réponse post-session du promoteur datée du 19/02/2015 incluant le formulaire d'information et de non opposition modifie – version 1.1 du 13/02/2015 -.

Lors de la séance du 20 JANVIER 2015

Après délibération, le Comité octroie un **AVIS FAVORABLE** à la recherche, aux motifs suivants :

L'étude apparaît pertinente et le rapport bénéfices/risques acceptable.
Les objectifs de la recherche sont définis et argumentés.
Les moyens mis en œuvre pour atteindre ces objectifs sont décrits avec suffisamment de précisions et apparaissent bien adaptés. La méthodologie est clairement décrite et adaptée aux objectifs.
Les notices d'information et formulaires de consentement sont clairement rédigés. Ils contiennent toutes les mentions nécessaires
Par ailleurs, le Comité s'approprie la motivation inscrite dans le courrier post-session du Président du 10/02/2015.

Ont participé à la délibération du 20 janvier 2015 :

Conformément aux dispositions de l'article R1123-11 du Code de la Santé Publique, ont participé à la délibération :

Collège n°1 :	Titulaires :	Suppléants :
Personnes qualifiées en matière de recherche biomédicale :	Dr. Baris TURAK	Pr. Guy MORIETTE (pédiatre)<br>Pr. Robin DHOTE
Pharmacien hospitalier :	Dr. Laurence ESCALUP (compétent en matière biostatistiques)	
Infirmier :	Bernadette SMUTEK	
Collège n°2 :		
Personnes qualifiées sur les questions éthiques :	Françoise KLELTZ-DRAPEAU	
Psychologue :	Nadine LABBE	
Personnes compétentes en matière juridique :	David SIMHON	
Représentant des associations agrées de malades et d'usagers du système de santé :	Paulette MORIN	

Le Président :

David Simhon

Committee for the Protection of Persons ILE DE FRANCE III

Composition of the Board

On 8 December 2014, the Committee received a request for an opinion on a routine care research project entitled ***SEPREVEN: Study on prevention and epidemiology of adverse events in neonates***

whose manager is: ***Centre Hospitalier Intercommunal de Créteil***

and the responsible person is: ***Laurence Caeymaex***

The research will take place in the following hospitals: ***CHI Creteil, CHU Grenoble, CH Pontoise, CHU R Debré, CH Angers, CHU Nice, CHU Bicetre, CHU Strasbourg, CHU Caen, CHU Lille, CH Poissy St germain, CH Delafontaine***

The Committee reviewed the research proposal (version1), the information and non-opposition form (version1) and all other documents provided by the sponsor, and took into account the sponsor's post-session response dated the same date, including the amended information and non-opposition form.

at the 20th of January 2015

After deliberation, the Committee delivers a favorable opinion on the research protocol on the following grounds:

- The study appears relevant and the benefit-risk ratio is acceptable

- The research objectives are defined and argued

- The means used to achieve the objectives are described in sufficient detail and appear to be appropriate. The methodology is clearly described and adapted to the objectives.

- Information notices and consent forms are clearly drawn up and contain all the necessary information

In accordance with the provisions of the article of the Public Health Code the following persons participated in this deliberation:

- person qualified in biomedical research matters Dr B TURAK
- hospital pharmacist Dr LESCALUP
- nurse Ms B SMUTEK
- persons qualified in ethical matters Ms F KLELTZ-DRAPEAU
- psychologist MS N LABBE
- legal professionals Mr D SIMHON
- representatives of approved associations of patients and users of the health system Ms P MORIN

The President, David SIMHON

Pr C. CHOUAID
Centre Hospitalier Intercommunal
de CRETEIL
Consultation de pneumologie
40 avenue de Verdun
94000 CRETEIL
Tél. : 01 57 02 20 70
Fax : 01 57 02 20 59
N° RPPS : 1000[illegible]54443

SEPREVEN : Protocol Amendments and Substantial Changes

- **Secondary objectives and related endpoints (added):** medico-economical analysis, skin tolerance after antisepsis, nasal tolerance during nasal ventilation; extubations/100 hours of intubation (accepted June 23, 2015); consequences of un-programmed extubations, characteristics of extravasation injuries, characterization of staphylococcus strains found in LOS; association of blood culture volume with growth time and positivity in sepsis; clinical characteristics of bacteraemia according to strains; association of workload and AE rates; Impact of intervention on safety culture **(accepted October 11, 2016)**

- **Estimation of the number of patients included in the entire SEPREVEN study (changed):** 6000 (instead of first estimation based on local investigators' estimation on patients stays in their unit 14000) **(accepted July 3, 2017)**

- **Inclusion criteria (changed)**: length of stay in the NICU was changed from ">= 48 hours" to "more than 2 days" **(accepted October 11, 2016)** for feasibility reasons, to avoid hour counts as an inclusion criteria.

Parental information and non-opposition and conduct of the research: "An exception will be made if the infant dies before the parents have received the information: the patient will then be included without contacting the parents after his decease, so as not to offend them." **(accepted June, 2017)**

« In cases where the infant dies before the parents can be informed, the child will be included in the study without parental information. Indeed, it does not seem conceivable to propose to parents who have just lost their child to participate in a study on the quality of care. Moreover, excluding these children would risk distorting the results and miss the analysis of potential serious adverse events in the care. **(accepted June, 2017)**

- **Retrospective chart review**: Practical description of the Trigger tool specified according to Paul Sharek's article and trigger toolkit; external, educated, reviewers not involved in the study; change in the number of charts to be analyzed: for financial reasons, due to the choice of using external reviewers, the number of charts had to be reduced from 20 to 15 per month per unit, for a total of 3600 instead of 4800 **(accepted July 3, 2017).**

- **Intervention (detailed description):** daily goal bundle, and poster to prevent extravasation injury **(accepted October 11, 2016)**.
